# Uniportal video-assisted thoracoscopic removal of a concealed percutaneously inserted sewing needle in the lung: A case report

**DOI:** 10.1097/MD.0000000000044366

**Published:** 2025-09-26

**Authors:** Yang Xu, Cheng Qian, Xin Chen, Jin-Hao Kou, Zhuang-Zhuang Cong, Jing Luo, Xiao-Long Liu, Yi Shen

**Affiliations:** a Department of Cardiothoracic Surgery, Jingling Hospital, Jingling School of Clinical Medicine, Nanjing Medical University, Nanjing, China; b Department of Cardiothoracic Surgery, Jingling Hospital, Medical School of Nanjing University, Nanjing, China; c Anesthesiology Department, Jingling Hospital, Medical School of Nanjing University, Nanjing, China.

**Keywords:** case report, intrapulmonary foreign body, percutaneous route, sewing needle, uniportal VATS

## Abstract

**Rationale:**

Percutaneous penetration of foreign bodies into the lung is exceptionally rare, with most reported cases involving entry through natural orifices such as the oral cavity or respiratory tract.

**Patient concerns:**

A 17-year-old adolescent was incidentally found to have a metallic sewing needle embedded in the right lung during imaging evaluation for unrelated symptoms. The foreign body had been retained for 9 years following a childhood needle-prick injury.

**Diagnoses:**

Imaging confirmed the presence of an intrapulmonary metallic foreign body consistent with a sewing needle.

**Interventions:**

Preoperative computed tomography–guided localization was performed to precisely identify the needle’s position, followed by successful surgical retrieval using uniportal video-assisted thoracoscopic surgery to remove the foreign body and surrounding affected tissue.

**Outcomes:**

The foreign body was completely removed with no intraoperative or immediate postoperative complications.

**Lessons:**

This case underscores the value of advanced imaging and minimally invasive surgical techniques in the management of rare intrapulmonary foreign bodies, particularly those retained for prolonged periods.

## 1. Introduction

While foreign bodies in the lungs are common in children,^[1,2]^ percutaneous penetration is far rarer than entry through natural orifices such as the oral or respiratory tract.^[3–6]^ Most cases involve ingestion or aspiration, whereas percutaneous introduction often goes undetected until incidental imaging reveals the anomaly.^[6]^ Sewing needles, an unusual subtype, are typically discovered during unrelated evaluations.^[7]^ These cases are noteworthy not only for their rarity but also for their risk of delayed complications, such as infections, lung abscesses, or vascular injury.^[8,9]^ Here, we report a 17-year-old adolescent with a concealed sewing needle retained in the lung for 9 years, which has detected incidentally during imaging. This report highlights the importance of advanced imaging and precise surgical techniques for effective management.

The use of minimally invasive uniportal video-assisted thoracoscopic surgery (VATS) for the removal of intrapulmonary foreign bodies is an innovative and effective approach. Uniportal VATS offers advantages over traditional multiportal approaches, including reduced trauma, faster recovery, and lower postoperative pain.^[10,11]^ Preoperative computed tomography (CT)-guided localization further enhances surgical precision by enabling accurate identification and removal of deeply embedded objects. This case underscores the importance of meticulous preoperative planning, advanced surgical methods, and interdisciplinary collaboration in managing rare intrapulmonary foreign bodies. The favorable outcome achieved here provides valuable insights into the potential of minimally invasive techniques to optimize treatment in similar scenarios.

## 2. Case report

A 17-year-old female adolescent was admitted to our hospital on September 11, 2024, with a concealed percutaneously inserted sewing needle in the right lower lobe of the lung. Three days before admission, the patient experienced recurrent left-sided chest pain and a chest X-ray revealed a linear metallic shadow in the right lower lung. Notably, the patient was unaware of the needle’s presence until imaging and expressed surprise at the finding. Upon detailed history-taking, she recalled an incident 9 years ago when she was pricked by a sewing needle while rolling on her bed, but no significant bleeding, swelling, or follow-up examination occurred. She believed that the needle had not penetrated the body and caused no harm, and was unaware of any potential risks or long-term complications.

**Figure 1.**
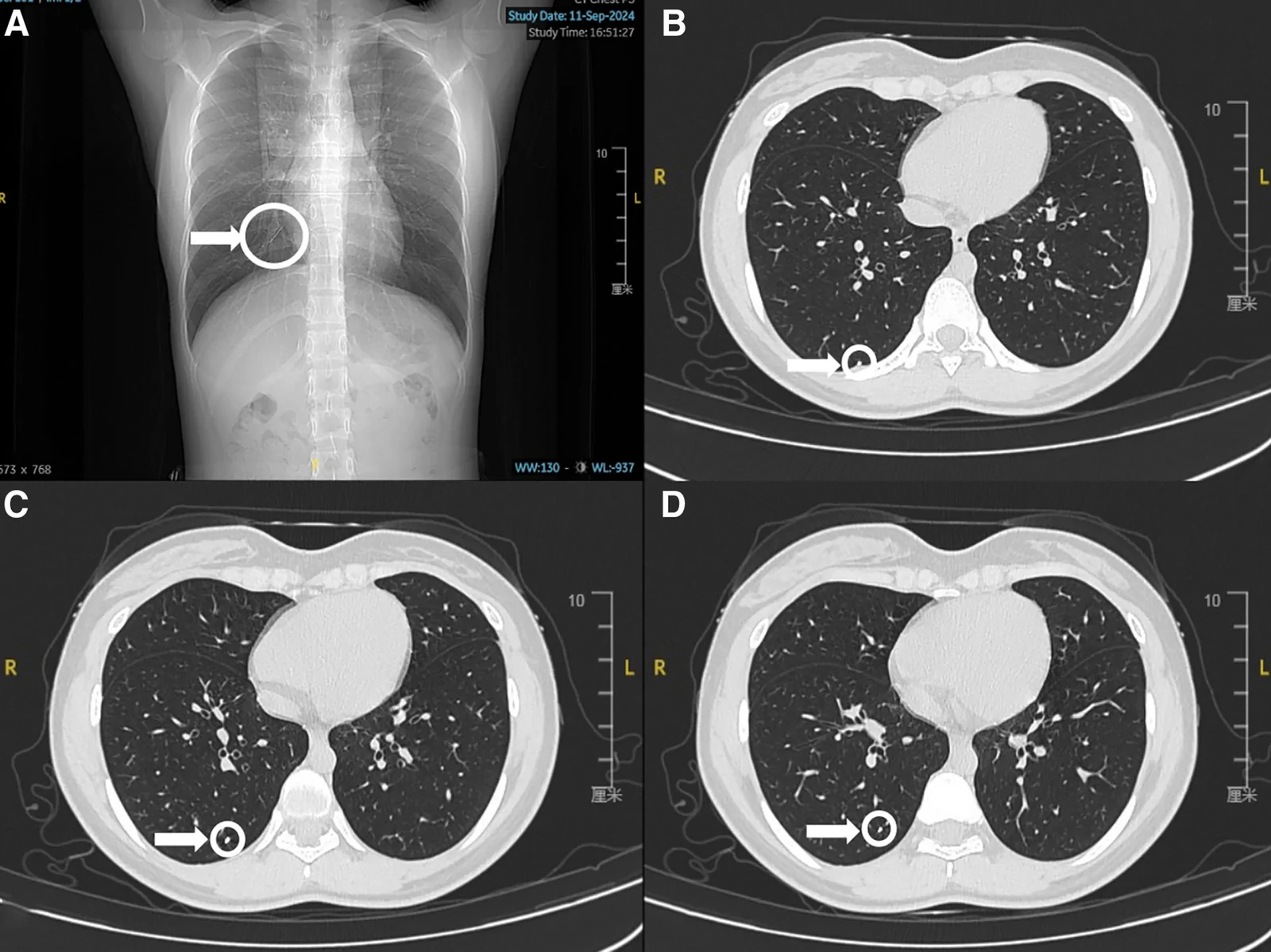
Preoperative radiological assessment. (A) Chest X-ray revealing a high-density foreign body in the right lower lung field (indicated by a white circle). (B–D) Axial thin-slice CT images demonstrating the trajectory of the sewing needle penetrating from the pulmonary surface to the deeper parenchymal tissue of the right lower lobe (white circles denote the sectional appearances of the sewing needle). CT = computed tomography.

After admission, CT confirmed a 23.3-mm-long, 2.8-mm-wide metallic foreign body in the posterior basal segment of the right lower lung, with no distinct chronic infection or granuloma formation around it on the CT image (Fig. 1). Blood tests, including white blood cell count (8.1 × 10⁹/L), neutrophil percentage (62.9%), and C-reactive protein (<0.5 mg/L), were within normal limits, ruling out active infection.

**Figure 2.**
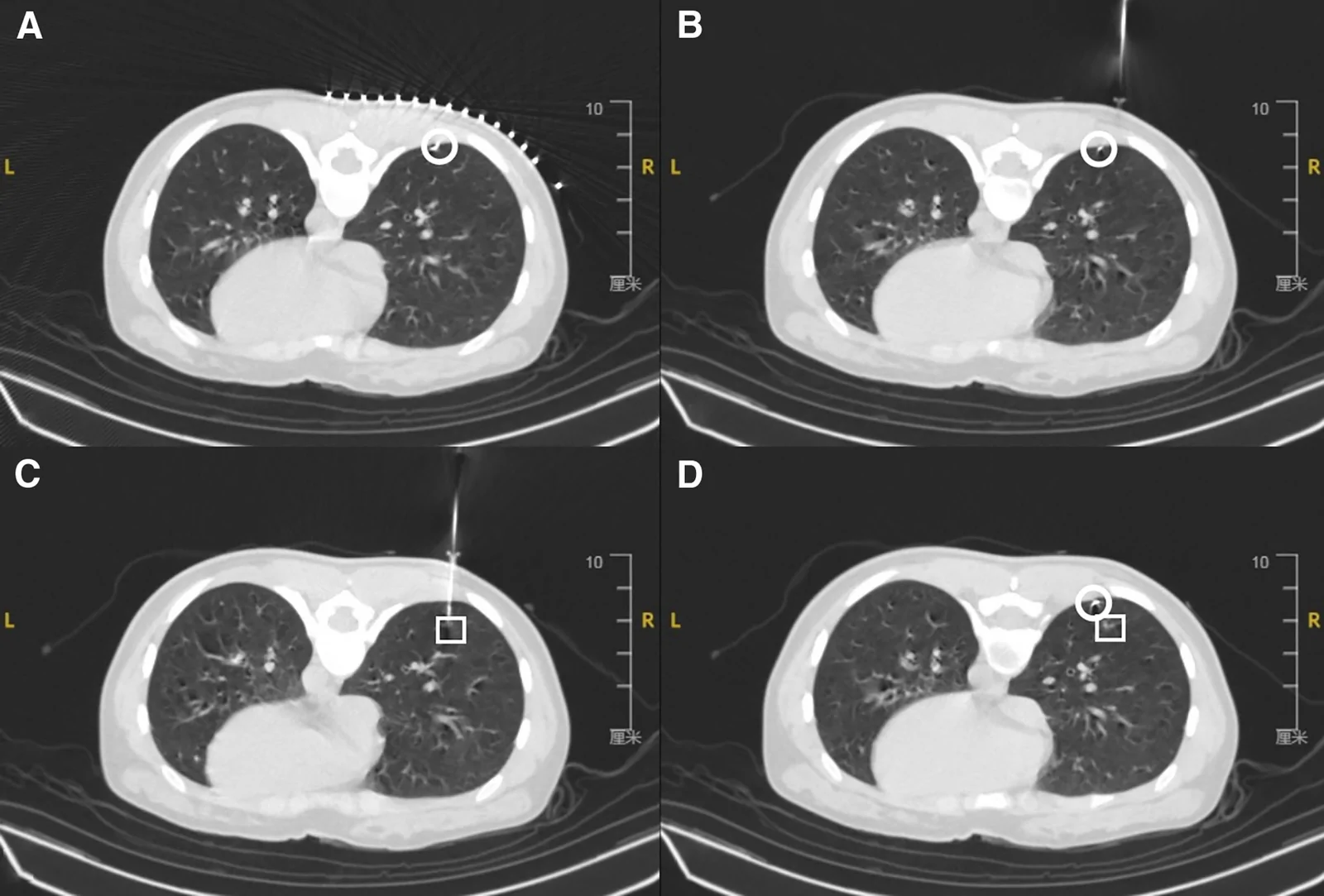
CT-guided preoperative localization of the pulmonary foreign body. (A) A positioning paper was placed on the patient’s back to assist in determining the needle puncture site. (B) After local anesthesia, the localization needle was inserted percutaneously into the thoracic cavity at the identified optimal site. (C) The localization needle tip was advanced near the foreign body within the lung. (D) A localization hookwire was deployed through the needle sheath to secure it in the lung near the foreign body, and the sheath was subsequently removed (white square highlights the localization hookwire, and white circle denotes the sewing needle). CT = computed tomography.

**Figure 3.**
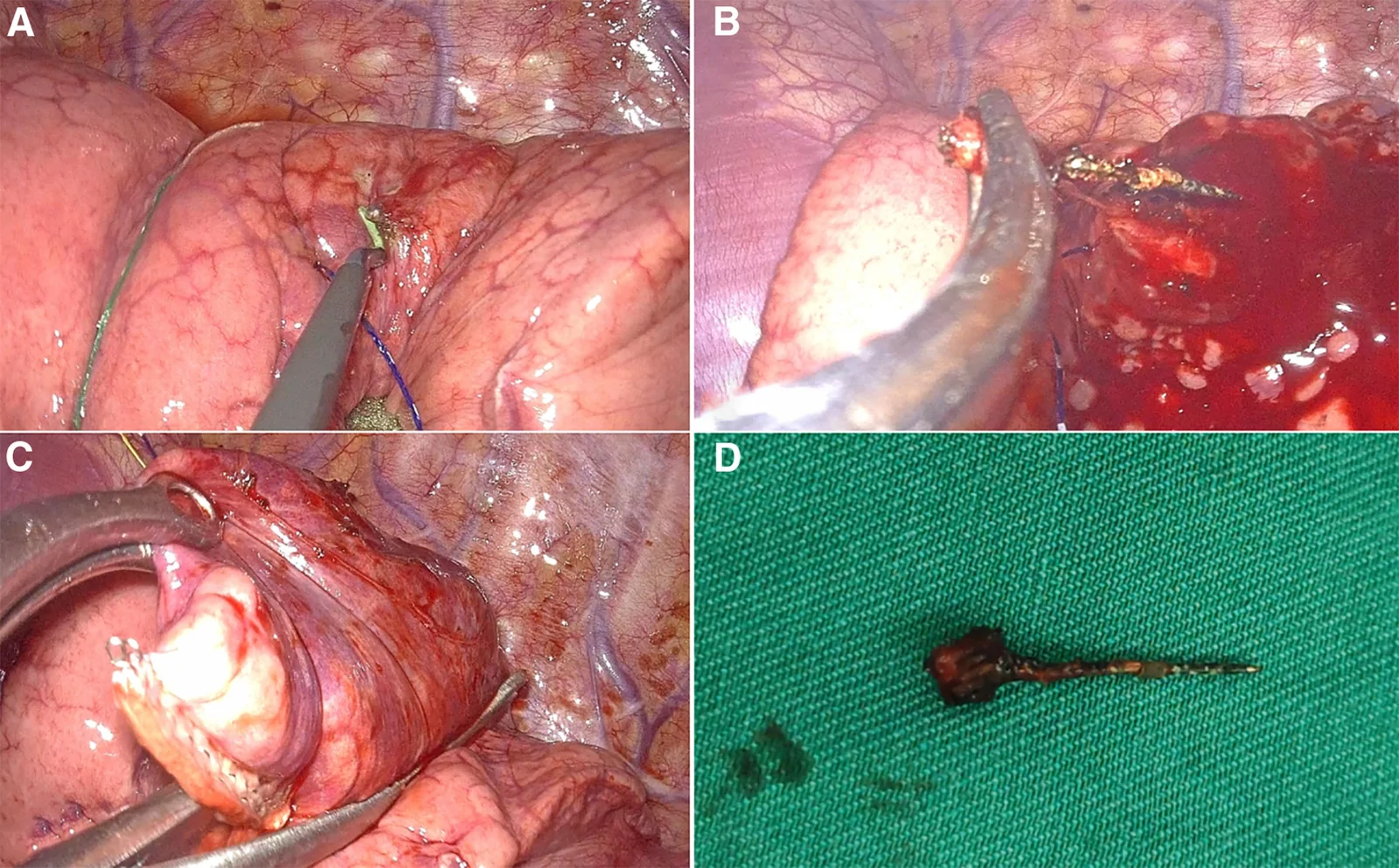
Uniportal VATS removal of sewing needle and resection of damaged lung tissue. (A) The visceral pleura covering the needle was opened near the blue location wire. (B) The sewing needle was extracted intact using a separating clamp. (C) Wedge resection of the damaged lung tissue and localization wire was performed. (D) The sewing needle retrieved from the lung. VATS = video-assisted thoracoscopic surgery.

A multidisciplinary meeting, including surgery, radiology, and pulmonology teams, considered that there was an indication for surgery. In order to achieve precise treatment, CT-guided localization was performed before minimally invasive surgery. A puncture needle was inserted ~5 mm from the foreign body under the guidance of CT, and the localization hookwire (featuring a 4-claw hook structure at its front to ensure secure anchoring within the lung tissue) was placed to guide surgical excision, taking a total of 10 minutes with no complications during the localization (Fig. 2). Wheelchair transport to the operating room helps avoid displacement of the localization wire. Uniportal VATS was then performed: a 3-cm incision was made at the 5th intercostal space, and a pleural protrusion could be seen beside the preoperative location site, in which the sewing needle was completely wrapped. To avoid needle fragmentation, the visceral pleura was initially opened, and the sewing needle was extracted slowly by separation plier to separate from the surrounding tissues, ensuring its integrity and no breakage (Fig. 3A and B). Wedge resection, including the destroyed lungs caused by the needle and the surrounding tissues that may be infected, ensured complete removal (Fig. 3C and D). Postoperative irrigation and air-leak testing confirmed no complications, and a single chest drain was placed. The operation was successfully completed through uniportal VATS, avoiding the conversion to open thoracotomy. The removed sewing needle was returned to the patient’s family, and the resected lung tissue was sent for pathological examination, which indicated chronic inflammation and alveolar congestion.

**Figure 4.**
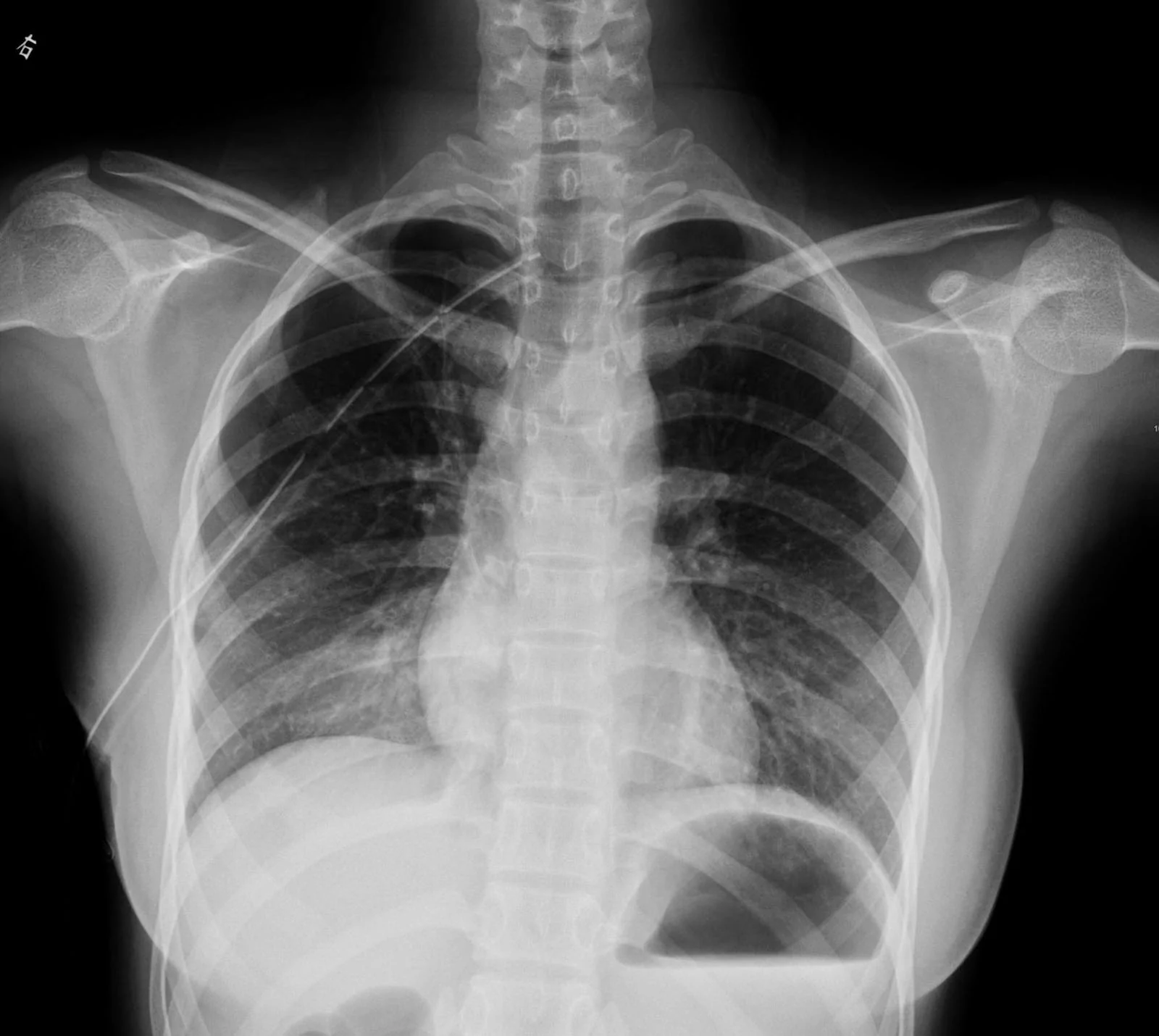
Postoperative day 1 chest X-ray demonstrates satisfactory lung re-expansion with no evidence of pneumothorax or pleural effusion. The foreign body shadow in the right lower lung has completely disappeared.

On postoperative day 1, a chest X-ray demonstrated satisfactory right lung re-expansion without residual foreign body, pneumothorax, or pleural effusion (Fig. 4). The chest drain was removed, and the patient was discharged the following day. At follow-up, she reported relief and had no symptoms, with imaging confirming resolution. No adverse events occurred. The report of the clinical case has been approved by the Ethics Committee of Jinling Hospital, and the patient and her family have signed the informed consent.

## 3. Discussion

This case of a sewing needle percutaneously retained in the lung for 9 years is exceedingly rare, as most intrapulmonary foreign bodies enter through natural orifices, while percutaneous penetration often goes unrecognized, leading to delayed presentation.^[1,3,12]^ The incidental discovery during imaging for unrelated chest pain aligns with prior reports where mild symptoms prompted evaluation.^[9]^ The patient’s vague recollection of the initial injury mirrors documented cases in which historical details were unclear, complicating diagnosis.

A key distinction of this case is its technical approach. Whereas prior studies emphasized imaging for localization,^[2]^ we applied preoperative CT-guided localization to ensure the accuracy. CT-guided localization has been routinely used in our center for biopsy of pulmonary tumor and preoperative marking of pulmonary nodules. In contrast to preoperative simulation or imaging reconstruction techniques, the precise placement of a localization wire adjacent to the foreign body eliminates the need for intraoperative exploration, significantly reducing both surgical time and anesthesia duration. This approach also facilitates more precise resection while better preserving healthy lung tissue during surgery. Preoperative CT-guided localization is minimally invasive and generally well-tolerated by patients. Wheelchair transport to the operating room helps avoid displacement of the localization wire. However, the potential use of intraoperative fluoroscopy might further reduce trauma associated with preoperative CT-guided localization – a hypothesis requiring further comparative clinical studies. In addition, previous studies have reported successful removal of an intrapulmonary needle under multi-port VATS,^[13]^ while no studies have reported the application of uniportal VATS in this setting. Compared with standard multiportal VATS, uniportal VATS offers reduced surgical trauma, less postoperative pain, and faster recovery.^[10,11,14]^ Furthermore, in the surgery, the sewing needle was first carefully extracted and then wedge resection of the damaged lung tissue and needle track was performed, assuring a complete removal. Our technique of CT-guided localization, uniportal VATS, removal of foreign body, as well as complete resection of the damaged lung may be helpful in the management of this condition.

The prolonged asymptomatic period and the absence of complications (e.g., lung abscess or vascular injury) further distinguish this case.^[4]^ Delayed presentation risks migration into critical structures, as seen in reports of needles puncturing the aorta,^[8]^ underscoring the need for early intervention. Metallic shadows on imaging warrant consideration of foreign bodies, tumors, or calcified granulomas, with thorough history and imaging being diagnostic cornerstones.

Prognosis is excellent with timely minimally invasive management, as demonstrated here. Preventive measures should emphasize the awareness of needle injuries and prompt evaluation, particularly in children. Integrating advanced imaging and surgical techniques into clinical practice may improve outcomes in such cases.

## Acknowledgments

We thank all the members of department of cardiothoracic surgery and anesthesiology department in our hospital that participated in this research.

## Author contributions

**Conceptualization:** Yang Xu, Cheng Qian.

**Data curation:** Yang Xu, Xin Chen, Yi Shen.

**Formal analysis:** Xin Chen, Xiao-Long Liu.

**Investigation:** Cheng Qian, Xin Chen, Xiao-Long Liu.

**Project administration:** Jing Luo, Yi Shen.

**Resources:** Jin-Hao Kou, Zhuang-Zhuang Cong, Jing Luo.

**Software:** Yang Xu, Jin-Hao Kou, Zhuang-Zhuang Cong.

**Supervision:** Zhuang-Zhuang Cong.

**Visualization:** Xiao-Long Liu.

**Writing – original draft:** Yang Xu, Cheng Qian.

**Writing – review & editing:** Zhuang-Zhuang Cong, Jing Luo, Yi Shen.
